# Revealing cerebrospinal fluid biomarkers in Parkinson's disease dementia based on iTRAQ proteomics research

**DOI:** 10.3389/fnins.2025.1682274

**Published:** 2025-11-07

**Authors:** Lin Han, Ying Liu, Changhong Tan, Lijuan Mo, Huahua Su, Ping Ma, Guotao Zeng, Jianhe Yue, Xi Liu, LiFen Chen

**Affiliations:** 1 Department of Neurology, The Second Affiliated Hospital of Chongqing Medical University, Chongqing, China; 2 Department of Neurology, West China School of Public Health and West China Forth Hospital, Sichuan University, Chengdu, China; 3 Department of Neurosurgery, The Second Affiliated Hospital of Chongqing Medical University, Chongqing, China

**Keywords:** proteomics, Parkinson disease dementia (PDD), cerebrospinal fluid (CSF), biomarker, isobaric tags for relative and absolute quantification (iTRAQ).

## Abstract

**Background:**

Parkinson's disease dementia (PDD) imposes a significant burden on patients and healthcare systems but currently lacks specific biomarkers. This study aimed to identify novel cerebrospinal fluid (CSF) biomarkers for PDD using proteomics and to explore their functional significance.

**Methods:**

Employing iTRAQ-based quantitative proteomics, we analyzed CSF from 73 participants included 34 PD patients, 14 PDD patients and and 25 healthy Controls (HCs). Bioinformatics analysis identified 44 differentially expressed proteins in PDD compared to PD (33 upregulated, 11 downregulated). Subsequent validation by ELISA confirmed a significant decrease in Insulin-like Growth Factor Binding Protein 3 (IGFBP3) concentration in PDD CSF compared to PD, while other candidates (NXPH1, LRRN1, HPRT1) showed no significant differences. *In vitro* functional studies using SH-SY5Y neuroblastoma cells demonstrated that IGFBP3 significantly attenuated cytotoxicity and apoptosis induced by the neurotoxin MPP^+^. IGFBP3 pretreatment improved cell viability (assessed by CCK-8 assay), reduced lactate dehydrogenase (LDH) release, and decreased apoptosis rates (measured by flow cytometry). Mechanistically, IGFBP3 counteracted MPP^+^-induced dysregulation of apoptosis markers (increased Bcl-2/Bax ratio; reduced cleaved caspase-3/caspase-3 ratio) and activated the PI3K/Akt/GSK3β signaling pathway by restoring phosphorylation levels of PI3K, Akt, and GSK3β.

**Results:**

These findings suggest that decreased CSF IGFBP3 is a potential biomarker for PDD. Furthermore, IGFBP3 exerts neuroprotective effects against MPP^+^ toxicity, likely mediated through the activation of PI3K/Akt/GSK3β pathway and inhibition of apoptosis.

**Conclusion:**

IGFBP3 warrants further investigation as a diagnostic biomarker and therapeutic target for PDD, necessitating future validation in larger cohorts and *in vivo* models.

## Introduction

Parkinson's disease (PD) is a common neurodegenerative disorder characterized by an insidious onset and a protracted clinical course. Its primary pathological hallmark is the degeneration of dopaminergic neurons in the substantia nigra pars compacta (Nwabufo and Aigbogun, 2022). Cardinal motor symptoms include bradykinesia, rigidity, resting tremor, and postural instability, while non-motor symptoms encompass cognitive impairment, mood disturbances, sleep disorders, and autonomic dysfunction (Xicoy et al., 2019). In advanced PD stages, worsening cognitive dysfunction significantly compounds motor disabilities, severely impacting quality of life and imposing a substantial burden on healthcare systems and families (Lawson et al., 2016). Parkinson's disease dementia (PDD) can manifest throughout the PD disease trajectory, with a higher prevalence in the later stages (van Breugel et al., 2025). Currently, the pathogenesis of PDD remains incompletely elucidated, and validated biomarkers are scarce. Although numerous biomarkers have been identified for PD itself, very few are specific to PDD (Parnetti et al., 2019).

Evidence from other neurodegenerative conditions, such as Alzheimer's disease (AD), indicates that diabetes mellitus increases the risk of developing AD (Yang et al., 2018). Recent perspectives suggest a potential link between PDD and insulin resistance, proposing that PDD patients may exhibit heightened susceptibility to it (Ashraghi et al., 2016). Furthermore, the frequent co-occurrence of Lewy body pathology in both PD/AD dementia suggests shared pathogenic mechanisms, with the insulin signaling pathway potentially playing a role in PDD development, because insulin signaling molecules have been shown to exert neuroprotective and proliferative functions by enhancing cell survival, preventing apoptosis, and stimulating neuronal development in areas such as the hippocampus, low expression of IGFBP3 may have affected the development and survival of neurons (Yang et al., 2018). Therefore, identifying diagnostic biomarkers for PDD, particularly those associated with the insulin signaling pathway, is of paramount importance.

Proteomic methodologies have matured into powerful tools for biomarker discovery (Rozanova et al., 2021). Among these, the isobaric tags for relative and absolute quantification (iTRAQ) technique is widely employed as a high-throughput assay for identifying protein biomarkers (Wen et al., 2014). In this study, we utilized an iTRAQ-based approach to profile differences in the cerebrospinal fluid (CSF) proteome between PD patients with and without dementia. Subsequent bioinformatics analysis and *in vitro* functional validation were performed to provide a theoretical foundation for the screening and clinical application of PDD CSF biomarkers.

## Methods and materials

### Clinical sample recruitment

Patients diagnosed with PD were recruited from the Department of Neurology at the Second Affiliated Hospital of Chongqing Medical University. Inclusion criteria were: (1) a diagnosis of PD according to the Movement Disorder Society (MDS) clinical diagnostic criteria (Postuma et al., 2015); (2) an age between 18 and 80 years. Exclusion criteria were: (1) a diagnosis of Parkinsonism, Parkinsonism-Plus syndromes, or other movement disorders and neurodegenerative diseases; (2) an inability to complete required questionnaires or undergo a lumbar puncture with physician assistance. Additionally, 25 healthy control participants (11 men and 14 women) without PD and other central nervous system disease were recruited with matched sex, age and education level. Cognitive status was assessed by two neurologists using the Mini-Mental State Examination (MMSE). The study protocol was approved by the Medical Ethics Committee of the Second Affiliated Hospital of Chongqing Medical University (Approval Number: 2020-189) and conducted in accordance with the ethical principles of the Declaration of Helsinki. Written informed consent was obtained from all participants prior to inclusion.

### CSF sample collection and processing

Approximately 2 mL of CSF was collected via lumbar puncture performed by experienced neurologists. Protease inhibitor (1 μL per 1 mL CSF; Protease Inhibitor Cocktail, Boyotime, China) was immediately added to each sample. CSF was collected in plain tubes and rapidly frozen at −80 °C within 30 min of collection.

### iTRAQ quantitative proteomics workflow

PD patients were classified into two groups based on MMSE scores: a PD without dementia group (MMSE < 27) and a PDD group (MMSE ≥ 27). Then, For mass spectrometry analysis, CSF samples from the PD, PDD, and HCs groups were labeled as Group A, B, and C, respectively. First, proteins were extracted and their concentration was quantified using the Bradford Protein Assay Kit (Bio-Rad, Richmond, USA). After electrophoresis, proteins were digested with trypsin at a 1:20 enzyme-to-substrate ratio. The resulting peptides were desalted, and freeze-dried. Peptide samples were dissolved in 0.5M TEAB and add it to the corresponding iTRAQ tag reagent. Different peptide segments were selected according to different samples iTRAQ labels. Second, labeled peptide samples were fractionated using a Shimadzu LC-20AB liquid phase system and C18 column for liquid phase pre-separation of samples (5 μm 4.6 × 250 mm Gemini), and then separated by Thermo UltiMate 3000 UHPLC. Finally, the Q-Exactive HF X (Thermo Fisher Scientific, San Jose, CA) tandem mass spectrometer was used to perform DDA (Data Dependent Acquisition) pattern detection.

### Bioinformatics analysis

Local Mascot server was used to against the corresponding databases and search for reliable protein identification results. iTRAQ-based quantitative analysis identified differentially expressed proteins based on fold change > 1.2 and Q-value < 0.05. Then, for identification of differentially expressed proteins (DEP), we firstly compared Group A (patients with PD without dementia) with Group C (HCs) to screen DEPs between PD without dementia patients and HCs, and compared Group B (patients with PDD) with Group C (HCs) to screen DEPs between PDD and HCs. These DEPs may reflect the difference between PD patients and HCs, and thus were excluded from further analysis. Then, Group A (patients with PD without dementia) were compared with Group B (patients with PDD) to screen the DEPs between PDD and PD. At last, abnormal *P*-values such as *p* = 0.049 and unique peptide number less than 3 were observed. Subsequent bioinformatics analyses included Gene Ontology (GO) enrichment, pathway enrichment (top 10 pathways), Eukaryotic orthologous group (KOG) functional annotation, cluster analysis, protein-protein interaction (PPI) network construction, and subcellular localization prediction.

### Enzyme-linked immunosorbent assay (ELISA)

Concentrations of IGFBP3, NXPH1, LRRN1, and HPRT1 in CSF were measured using human ELISA kits (IGFBP3: CSB-E04590h, CUSABIO, China; NXPH1: ELK3869, ELK Biotechnology, China; LRRN1: MBS2602720, MyBioSource, USA; HPRT1: ELK3546, ELK Biotechnology, China). Assays were conducted according to manufacturers' protocols. Absorbance values of standard and sample samples were measured using an ELISA reader at 450 nm by spectrophotometry.

### Cell culture and treatment

Human neuroblastoma SH-SY5Y cells (CL-0208, Procell, Wuhan, China) were cultured in DMEM/F12 medium (Gibco, USA) supplemented with 10% fetal bovine serum (FBS; CTCC-001-071, Meisencell, China) at 37 °C under 5% CO_2_. The medium was refreshed every 2–3 days. Based on preliminary CCK-8 assays, the optimal concentrations for subsequent experiments were determined to be 1 mM for 1-methyl-4-phenylpyridinium (MPP^+^; D048, Sigma, USA) and 100 ng/mL for IGFBP3. Cells were assigned to four treatment groups: (1) Control (medium only, 48 h); (2) MPP^+^ (exposed to 1 mM MPP^+^ for the final 24 h); (3) MPP^+^ + IGFBP3 (pretreated with 100 ng/mL IGFBP3 for 24 h, then co-treated with MPP^+^ for 24 h); and (4) IGFBP3 (100 ng/mL IGFBP3, 48 h).

### Cell viability assay (CCK-8)

SH-SY5Y cells were seeded in 96-well plates at a density of 5 × 10^3^ cells/well (*n* = 3 wells per group). After 48 h, 10 μL of CCK-8 solution (C0037, Beyotime, China) was added to each well. Following a 2-h incubation, absorbance was measured at 450 nm using a microplate reader.

### Lactate dehydrogenase (LDH) release assay

Cytotoxicity was assessed by measuring LDH activity released from damaged cells using an LDH Cytotoxicity Assay Kit (C0018S, Beyotime, China) with WST-8. SH-SY5Y cells were seeded in 96-well plates. After 48 h of treatment, the assay was performed according to the kit instructions. Absorbance was measured at 450 nm to calculate LDH release.

### Flow cytometry for apoptosis

Apoptosis was detected using an Annexin V-FITC/PI Apoptosis Detection Kit (C1062S, Beyotime, China). Briefly, 1 × 106 SH-SY5Y cells were seeded in 6-well plates and treated for 48 h. Cells were centrifuged (1,000 rpm, 5 min), washed twice with PBS, resuspended in 500 μL binding buffer, stained with 5 μL Annexin V-FITC and 10 μL propidium iodide for 15 min in the dark at room temperature, and analyzed immediately by flow cytometry (CytoFLEX, Beckman, USA).

### Western blot analysis

Cellular protein was extracted and quantified using a BCA Protein Assay Kit (GK10009, GLPBIO, USA). Samples (30 μg protein per lane) were mixed with loading buffer (1:4 dilution), denatured (95 °C, 10 min), separated by SDS-PAGE (80V stacking, 120V resolving), and transferred onto polyvinylidene difluoride (PVDF) membranes at 250 mA constant current (transfer time adjusted based on protein molecular weight, 30–90 min). Membranes were blocked with Protein-Free Rapid Blocking Buffer (PS108P, Epizyme, China) for 10 min, washed three times with TBST (5 min each), and incubated overnight at 4 °C with primary antibodies against: PI3K (1:1,000, #4292, CST, USA), Phospho-PI3K (1:1,000, #17366, CST, USA), Akt (1:1,000, #9272, CST, USA), Phospho-Akt (1:1,000, #9271, CST, USA), GSK3β (1:1,000, #12456, CST), Phospho-GSK3β (1:1,000, #5558, CST, USA), Bcl-2 (1:1,000, T40056, Abmart, China), Bax (1:1,000, T40051, Abmart, China), Caspase-3 (1:1,000, T40044, Abmart, China), Cleaved Caspase-3 (1:1,000, #9661, CST, USA), β-Actin (1:20,000, #66009-1-Ig, Proteintech, China). After washing (3 × TBST, 5 min each), membranes were incubated with appropriate secondary antibodies (room temperature, 1 h). Protein bands were visualized using chemiluminescence and quantified by densitometry using ImageJ software (National Institutes of Health, Bethesda, Maryland, USA).

### Statistical analysis

Numerical variable data presented were expressed as mean and standard deviation (Mean ± SD). Multiple comparisons between groups were performed by one-way ANOVA with Tukey's *post hoc* tests or Welch's ANOVA with Tamhane's T2 post hoc tests and unpaired Student's *t*-test to analyze differences between two groups. Categorical variable data were expressed as percentages, and intergroup differences were assessed using chi-squared or Fisher's exact tests. All statistical analyses were performed using GraphPad Prism 9.5 (GraphPad software, CA, USA). *P* value of < 0.05 was considered statistically significant.

## Results

### Demographic and clinical characteristics of subjects

The study cohort comprised 73 participants: 34 with PD, 14 with PDD, and 25 HCs. The mean age was comparable across all groups (PD: 62.21 ± 8.53 years; PDD: 64.21 ± 7.30 years; HCs: 60.24 ± 5.47 years). Disease duration was longer in the PDD group than that in the PD group (4.91±5.85 vs. 4.11±3.82 years). A statistically significant difference in MMSE scores was observed among the three groups (PD: 28.29 ± 1.38; PDD: 20.64 ± 4.25; HCs: 29.21 ± 1.11, *p* < 0.0001). In addition, there was no statistically significant difference in age, gender, or education level among the three groups (Table 1).

**Table 1:** Basic characteristics and scale scores of the subjects.

**Clinical characteristics and scale scores**	**Parkinson disease (*n* = 34)**	**Parkinson disease dementia (*n* = 14)**	**HCs (*n* = 25)**	***P***
Gender (*N*, % men)	22 (64.71%)	6 (42.86%)	11 (44%)	0.196
Age, mean ± SD, years	62.21 ± 8.53	64.21 ± 7.30	60.24 ± 5.47	0.2661
Educational level				0.251
Junior-middle school or below, *N* (%)	20 (58.82)	12 (85.71)	20 (80)	
High school, *N* (%)	9 (26.47)	1 (7.14)	4 (16)	
Bachelor's degree or above, *N* (%)	5 (14.71)	1 (7.14)	1 (4)	
Disease duration, mean ± SD, years	4.11 ± 3.82	4.91 ± 5.85		0.5784
MMSE, mean ± SD	28.29 ± 1.38	20.64 ± 4.25	29.21 ± 1.11	< 0.0001

### iTRAQ-Based CSF proteomic profiling

After converting the raw mass spectrometry data into an mgf format file through Proteome Discoverer (ThermoFisher Scientific, USA), the corresponding protein sequence database was searched and identified using protein identification software Mascot 2.3.02(Matrix Science Ltd, UK). At the same time as quality control analysis, determine whether the data in this case was qualified. After passing a certain screening threshold after the data was qualified, the final reliable protein identification result was obtained. Subsequently, iTRAQ quantitative analysis was performed, and CSF samples from PD, PDD, and HCs groups were labeled as groups A, B, and C, respectively. One repeated experiment was conducted, and a total of 8,85,123 secondary spectra were generated. Under the “1% FDR” filtering standard, a total of 7,987 peptide segments and 1,531 proteins were identified. In the case of repeated experiments, quantitative repeatability analysis was used to determine whether the quality of the quantitative data meets the standard. The significantly different proteins in a single experiment were screened under two conditions: fold change>1.2 and Q-value < 0.05. Then, intra group comparisons of PD, PDD, and HCs were conducted to identify PDD related differentially expressed proteins, including 34 upregulated proteins and 11 downregulated proteins (Tables 2, 3).

**Table 2:** Upregulated CSF proteins in PDD group compared with PD group.

**Entry**	**Protein names**	**Gene names**	**Log2 (Fold change)**
Q6UXK5	Leucine-rich repeat neuronal protein 1	LRRN1	0.454176
P58417	Neurexophilin-1	NXPH1	0.432959
P00492	Hypoxanthine-guanine phosphoribosyltransferase	HPRT1	0.400538
Q9UBQ6	Exostosin-like 2	EXTL2	0.378512
Q9Y639	Neuroplastin	NPTN	0.378512
Q2TAL6	Brorin	VWC2	0.367371
P41222	Prostaglandin-H2 D-isomerase	PTGDS	0.367371
O95390	Growth/differentiation factor 11	GDF11	0.356144
P22692	Insulin-like growth factor-binding protein 4	IGFBP4	0.356144
Q9UKU6	Thyrotropin-releasing hormone-degrading ectoenzyme	TRHDE	0.344828
P08572	Collagen alpha-2(IV) chain	COL4A2	0.333424
P24593	Insulin-like growth factor-binding protein 5	IGFBP5	0.333424
P08670	Vimentin	VIM	0.333424
Q9UMF0	Intercellular adhesion molecule 5	ICAM5	0.321928
Q9Y5I4	Protocadherin alpha-C2	PCDHAC2	0.321928
Q10469	α-1,6-mannosyl-glycoprotein 2-β-N-acetylglucosaminyltransferase	MGAT2	0.321928
P10451	Osteopontin	SPP1	0.31034
Q8NBJ4	Golgi membrane protein 1	GOLM1	0.31034
P16930	Fumarylacetoacetase	FAH	0.31034
Q86VZ4	Low-density lipoprotein	LRP11	0.298658
O00339	Matrilin-2	MATN2	0.298658
P14314	Glucosidase 2 subunit beta	PRKCSH	0.298658
P51693	Amyloid-like protein 1	APLP1	0.298658
Q99574	Neuroserpin	SERPINI1	0.298658
Q86VZ4	Receptor-related protein 11		0.298658
Q13449	Limbic system-associated membrane protein	LSAMP	0.286881
P30530	Tyrosine-protein kinase receptor UFO	AXL	0.286881
O00391	Sulfhydryl oxidase 1	QSOX1	0.286881
Q6UWP8	Suprabasin	SBSN	0.286881
P04075	Fructose-bisphosphate aldolase A	ALDOA	0.275007
Q99985	Semaphorin-3C	SEMA3C	0.275007
P02649	Apolipoprotein E	APOE	0.275007
Q9BY67	Cell adhesion molecule 1	CADM1	0.275007

**Table 3:** Downregulated CSF proteins in PDD group compared with PD group.

**Entry**	**Protein names**	**Gene names**	**Log2 (Foldchange)**
O95445	Apolipoprotein M	APOM	−0.434402824
P35527	Keratin, type I cytoskeletal 9	KRT9	−0.434402824
O60888	Protein Cut A	CUTA	−0.340075442
P02774	Afamin	AFM	−0.340075442
P0DOX8	Immunoglobulin lambda-1 light chain		−0.340075442
P01042	Kininogen-1	KNG1	−0.321928095
P17936	Insulin-like growth factor-binding protein 3	IGFBP3	−0.304006187
P07357	Complement component C8 alpha chain	C8A	−0.268816758
P05156	Complement factor I	CFI	−0.268816758
P04003	C4b-binding protein alpha chain	C4BPA	−0.268816758
P00751	Complement factor B	CFB	−0.152003093

### Bioinformatics analysis of differentially expressed proteins

**Figure 1:**
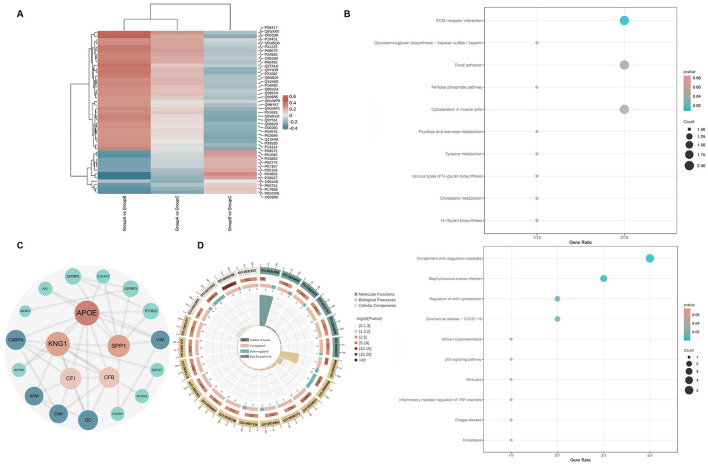
Bioinformatic analysis of differentially expressed proteins in CSF. **(A)** Heatmap of differentially expressed proteins in PDD vs. PD (Fold change >1.2, Q-value < 0.05). **(B)** Top 10 enriched KEGG pathways for upregulated and downregulated differentially expressed proteins. **(C)** Protein-protein interaction (PPI) network of differentially expressed proteins. **(D)** Gene Ontology (GO) functional annotation of differentially expressed proteins.

To elucidate the biological relevance of the differential expressed proteins in PDD pathogenesis, we performed KOG functional annotation, top 10 pathway enrichment analysis, and protein interaction network analysis (Figure 1). Firstly, based on functional analysis, whether proteins related to hippocampal structure, such as LRRN1, promoted synaptic formation, development, and regeneration; NXPH: promoted the formation of dendritic and axonal adhesion proteins; whether it affected neuronal function, such as HPRT1, which is mostly distributed in the basal ganglia and may affected dopaminergic neuron function, and IGFBP3, which may played a role in the development of PDD through the insulin signaling pathway. Secondly, proteins were screened based on their differential fold and protein interaction relationships. Finally, based on whether the CSF sample was within the detection range of the ELISA kit, four candidate proteins were ultimately determined (three upregulated: NXPH1, LRRN1, HPRT1; one downregulated: IGFBP3) for ELISA validation.

### PDD patients exhibit decreased CSF IGFBP3 level compared to PD patients

**Figure 2:**
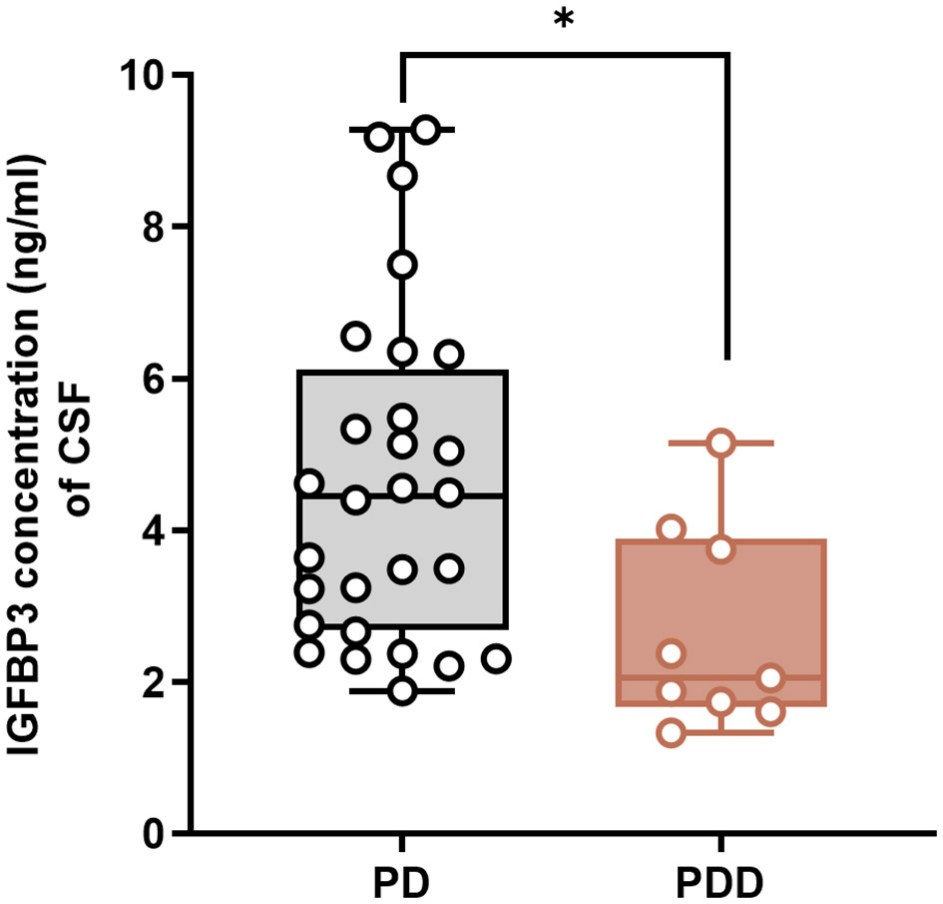
PDD patients had decreased CSF IGFBP3 level compared to PD patients. ELISA results of IGFBP3 detection showed that IGFBP3 concentration was lower in the CSF of PDD patients than PD patients (*n* = 28 in PD group and *n* = 9 in PDD group). Columns with error bars present the mean ± SEM. Two-tailed unpaired *t*-tests. *p < 0.05.

To clarify the changes in four target proteins in CSF of PD and PDD groups, ELISA was performed on CSF samples from the entire cohort (28 PD and 9 PDD) to correlate proteomic findings with clinical status. Partial missing data were due to the patient's withdrawal midway, resulting unable to obtain CSF samples or were unable to use them due to storage or severe blood contamination. Concentrations of NXPH1, LRRN1, and HPRT1 showed no significant differences between PD and PDD groups (*p* = 0.742, *p* = 0.149, *p* = 0.455, respectively). In contrast, IGFBP3 concentration was significantly decreased in the PDD group compared to the PD group (*p* = 0.0161; Figure 2). Based on these results, it was suggested that IGFBP3 may be involved in the neuropathological process of PDD, affecting the survival of neurons, IGFBP3 was prioritized as a potential PDD biomarker candidate for further functional investigation *in vitro*.

### IGFBP3 attenuates MPP^+^-induced viability and cytotoxicity in SH-SY5Y cells

**Figure 3:**
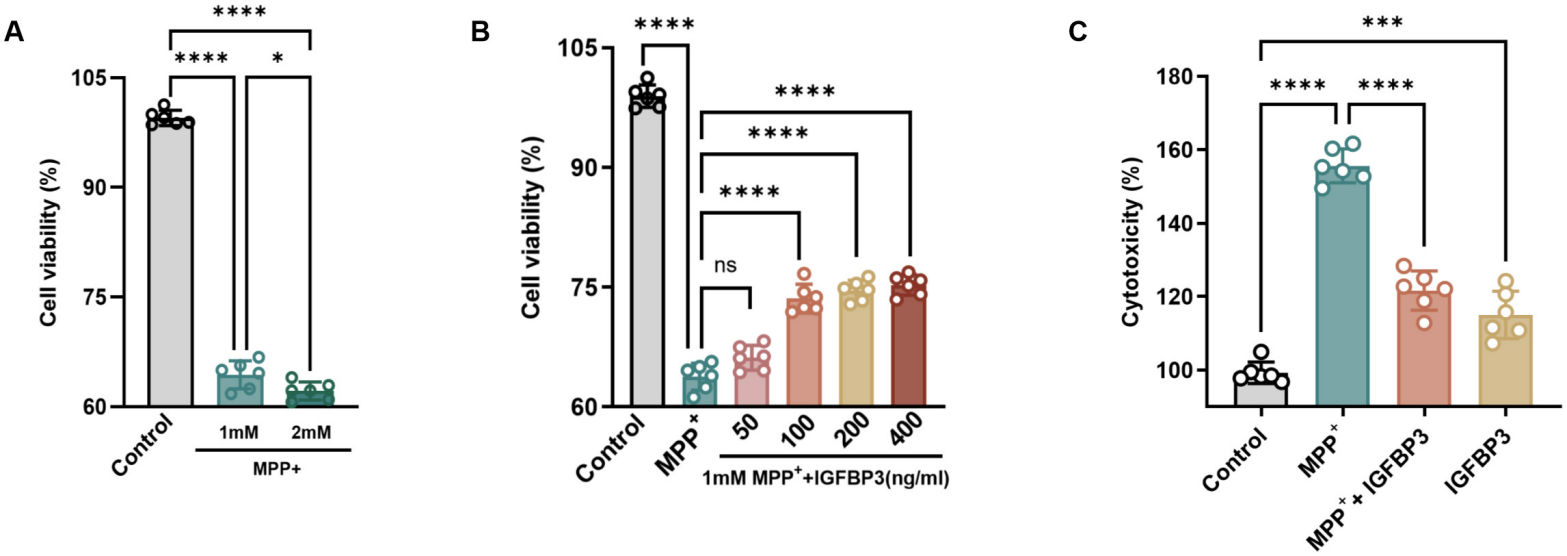
IGFBP3 Attenuates MPP^+^-induced viability and cytotoxicity in SH-SY5Y cells. **(A)** Effects of MPP^+^ at different concentrations on SH-SY5Y cell viability detected by CCK-8 assay (*n* = 6). **(B)** Effects of IGFBP3 at different concentrations on SH-SY5Y cell viability detected by CCK-8 assay (*n* = 6). **(C)** Cytotoxicity of 100 ng/mL IGFBP3 treatment and 1 mM MPP^+^-introduced in SH-SY5Y cells in each group was determined by LDH assay (*n* = 6). Columns with error bars present the mean ± SEM. one-way ANOVA with Tukey's *post hoc* tests **(A–C)** ^ns^*p* > 0.05, *p < 0.05, ***p < 0.001, and ****p < 0.001.

To clarify the effect of IGFBP3 on neuronal survival, the CCK8 assay was used to analyze MPP^+^-induced survival rate of SH-Y5Y cells. The CCK8 assay results showed that SH-SY5Y cell viability changes were induced by MPP^+^ at different concentrations (1 and 2 mM), and the optimal concentration of MPP^+^ was ultimately determined to be 1 mM based on cell survival rate (Figure 3A). At the same time, different concentrations of IGFBP3 (50, 100, 200, and 400 ng/mL) were added. It can be seen that the cell survival rates were 65.66, 73.77, 74.86, and 75.18%, respectively. Except for 50 ng/mL IGFBP3 treatment, compared with the 1 mM MPP^+^ group, 100, 200, and 400 ng/mL IGFBP3 significantly rescued the cell survival rate. However, inter-group comparisons showed high concentration IGFBP3 (200, 400 ng/mL) did not induce higher cell survival rate following 1 mM MPP^+^ treatment. Therefore, 100 ng/mL IGFBP3 was selected as the optimal concentration for subsequent experiments (Figure 3B). To clarify the effect of IGFBP3 on neuronal survival, the lactate dehydrogenase (LDH) assay was used to analyze MPP^+^-induced cytotoxicity of SH-Y5Y cells. Consistent with this, the MPP^+^-induced increase in LDH release was significantly attenuated by IGFBP3 pretreatment in SH-SY5Y cells. Compared with the 1 mM MPP^+^ group, the LDH activity in 1 mM MPP^+^ +100 ng/mL IGFBP3 group decreased from 159.09 to 120.2% (*p* = 0.0003) (Figure 3C).

### IGFBP3 inhibited MPP^+^-induced apoptosis in SH-SY5Y cells

**Figure 4:**
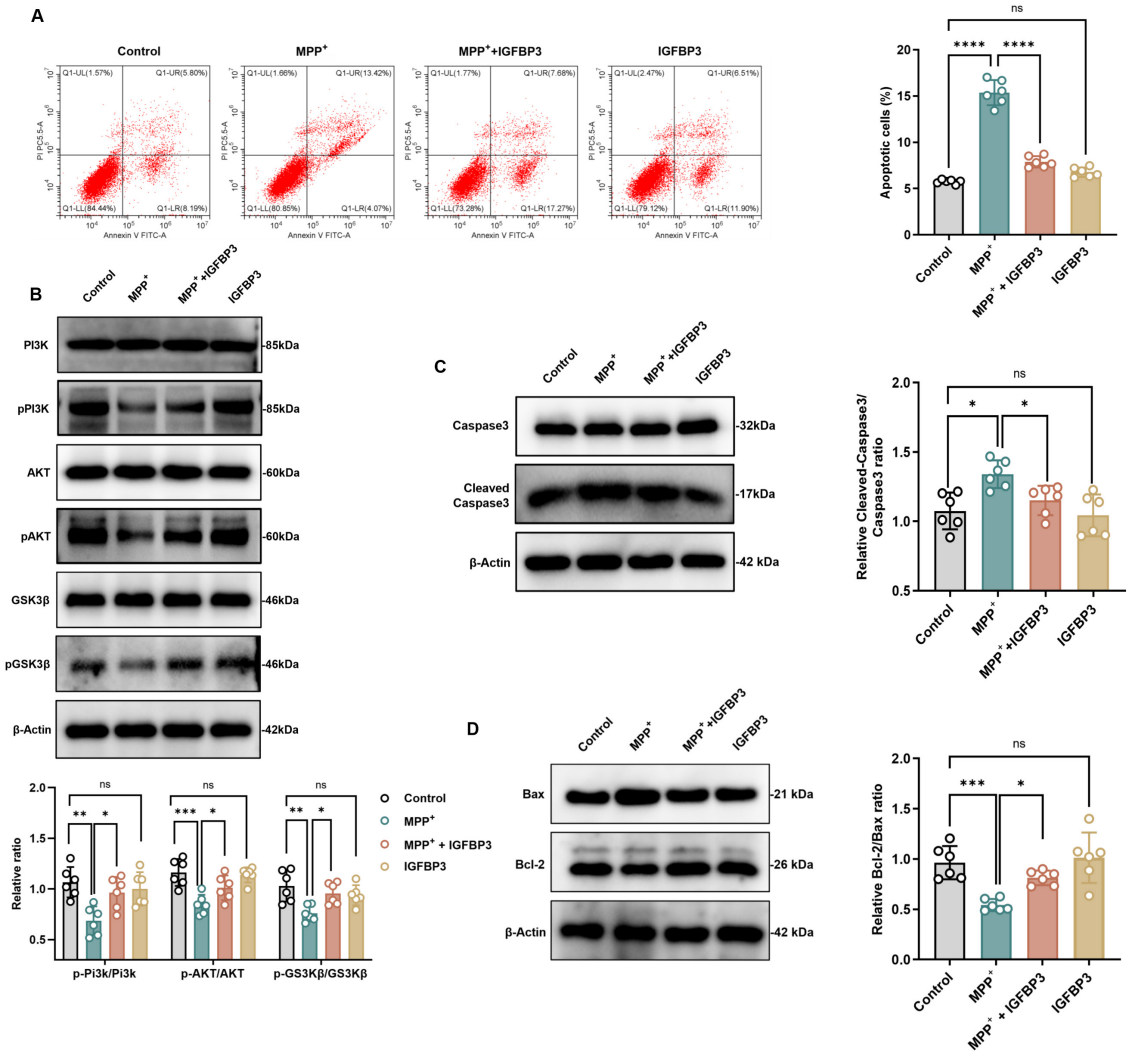
IGFBP3 inhibited MPP^+^-induced apoptosis and activated the PI3K/Akt/GSK3β pathway in SH-SY5Y cells. **(A)** Flow cytometry results of cell apoptosis in SH-SY5Y cells (*n* = 6). **(B)** Western blot and quantitative analysis of phosphorylation/total protein ratio of PI3K/AKT/GSK3β in SH-SY5Y cells (*n* = 6). **(C, D)** Western blot and quantitative analysis of cleaned-caspase3, caspase3, Bcl-2 and Bax in SH-SY5Y cells (*n* = 6). Columns with error bars present the mean ± SEM. one-way ANOVA with Tukey's *post hoc* tests (Flow cytometry, Bcl-2/Bax ratio, pAKT/AKT ratio, pGSK3β/GSK3β ratio). Welch's ANOVA with Tamhane's T2 *post hoc* tests (Cleaved-Caspase3/Caspase3 ratio, pPi3k/Pi3k ratio). ^ns^ *p* > 0.05, *p < 0.05, **p < 0.01, ***p < 0.001, and ****p < 0.001.

To clarify whether IGFBP3 has the effect of reducing cell apoptosis, we used flow cytometry to observe the situation of cell apoptosis. Flow cytometry analysis confirmed that IGFBP3 pretreatment significantly reduced the apoptosis rate induced by 1 mM MPP^+^ (13.42% 1 mM MPP^+^ vs. 7.68% 1 Mm MPP^+^ + IGFBP3, *p* < 0.0001; Figure 4A). IGFBP3 alone had minimal effect on apoptosis. We also detected apoptosis related biomarkers through western blot assay, including the anti-apoptotic member Bcl-2 and the pro-apoptotic member Bax of the Bcl-2 family and caspase 3 of the caspase family. Their expression levels are important indicators for evaluating apoptosis, and we also need to detect the expression levels of cleaved caspase protein activators, namely cleaved caspase. 1 mM MPP^+^ treatment decreased the anti-apoptotic protein Bcl-2, increased the pro-apoptotic protein Bax, and consequently reduced the Bcl-2/Bax ratio. IGFBP3 pretreatment reversed these changes, increasing Bcl-2, decreasing Bax, and restoring the Bcl-2/Bax ratio (*p* = 0.0045; Figure 4D). Similarly, 1 mM MPP^+^ increased the cleaved caspase-3/caspase-3 ratio, indicative of caspase-3 activation. IGFBP3 pretreatment significantly attenuated this increase (*p* = 0.0369; Figure 4C), indicating inhibition of the apoptotic execution phase.

### IGFBP3 activates the PI3K/Akt/GSK3β signaling pathway in SH-SY5Y cells

To explore the neuroprotective mechanism underlying IGFB3′s neuroprotective effects, we examined its effect on the PI3K/Akt/GSK3β pathway, known to regulate cell survival and apoptosis. Western blot analysis revealed that MPP^+^ treatment significantly decreased the phosphorylation levels of PI3K, Akt, and GSK3β. IGFBP3 pretreatment effectively restored the phosphorylation levels of all three kinases (Figure 4B). Treatment with IGFBP3 alone had negligible effects on the phosphorylation status of these proteins.

## Discussion

Historically, research and treatment for PD have predominantly focused on motor symptoms, often overlooking non-motor manifestations such as cognitive decline. Consequently, PDD was frequently misattributed to normal aging or confused with other dementias (Xu et al., 2025). With the increasing awareness of PD in recent years and the heavy burden it brings to families and society, researchers have gradually paid attention to it. However, many PDD patients are diagnosed in the late stage, associated with severe cognitive decline, inability to take care of themselves, and poor treatment effects (Abbood et al., 2024). This underscores the critical need for PDD-specific biomarkers. Although CSF sampling is less accessible than serum, its proximity to the central nervous system makes it an ideal source for neurodegenerative disease biomarkers (Parnetti et al., 2019). Utilizing iTRAQ proteomics, we identified a significant decrease in CSF IGFBP3 levels in PDD patients compared to PD patients without dementia. Our *in vitro* studies demonstrated that IGFBP3 protects SH-SY5Y cells against MPP^+^-induced cytotoxicity and apoptosis, potentially via activation of the PI3K/Akt/GSK3β signaling pathway.

The insulin-like growth factor system, particularly IGFBP3, is implicated in age-related degenerative disorders, influencing neuronal proliferation, differentiation, apoptosis, and synaptic plasticity (Dai et al., 2017). Supporting this, prospective studies link circulating IGFBP3 levels to age-related cognitive domains (e.g., memory, processing speed) and white matter hyperintensity volume (Salzmann et al., 2021). Reduced plasma IGFBP-3 levels have been observed in AD patients compared to controls (Ma et al., 2015). Furthermore, PD patients with comorbid diabetes exhibit more pronounced attentional and executive dysfunction than those without diabetes (Ashraghi et al., 2016). Mice with IGFBP3 gene deletions display impaired neuronal structure, signal transduction, and spatial working memory (Dai et al., 2017). Cumulatively, these findings suggest insulin/IGF signaling dysfunction contributes to the pathogenesis of aging and neurodegenerative diseases like AD and PDD. Our CSF proteomic analysis and bioinformatics findings, coupled with functional validation, support the hypothesis that decreased IGFBP3 is involved in PDD pathogenesis, potentially sharing mechanisms with AD and age-related cognitive decline.

Accumulating evidence indicates IGFBP3 possesses cytoprotective properties, promoting cell survival, proliferation, and differentiation (Salzmann et al., 2021), aligning with our results. We also observed that the protective efficacy of IGFBP3 against MPP^+^ toxicity was concentration-dependent within a specific range (100–200 ng/mL), beyond which no proportional increase in survival occurred. This finding may inform future biomarker development and therapeutic dosing strategies.

The PI3K/Akt/GSK3β pathway is a critical regulator of cell survival and apoptosis in neurodegenerative contexts (Yue et al., 2017). Akt, activated by PI3K, translocates to mitochondria upon stress and phosphorylates/inactivates GSK3β at Ser9. Phosphorylated/inactive GSK3β promotes cell survival, while its active form promotes apoptosis (Zhang et al., 2016). Our data demonstrate that IGFBP3 counteracts 1 Mm MPP^+^-induced dephosphorylation (inactivation) of PI3K/Akt as well as the dephosphorylation (activation) of GSK3β, thereby activating this pro-survival cascade.

Apoptosis is executed via caspase activation, regulated by the Bcl-2 family (e.g., Bcl-2 anti-apoptotic, Bax pro-apoptotic) (Liu et al., 2013; Lee et al., 2012). An imbalance favoring Bax triggers the mitochondrial apoptotic pathway, culminating in caspase-3 activation (Liu et al., 2013; Warren et al., 2019). Our results show that IGFBP3 restores the 1mM MPP^+^-induced imbalance in Bcl-2/Bax expression and reduces cleaved caspase-3 levels, thereby inhibiting execution of apoptosis and enhancing neuronal survival.

While our findings are derived from a modest clinical sample size, the consistent results across proteomics, ELISA, and multiple *in vitro* functional assays strongly suggest IGFBP3 as a potential PDD biomarker candidate. Future validation in larger cohorts and *in vivo* models is warranted.

## Conclusion

To identify novel biomarkers for PDD, we employed iTRAQ proteomics to analyze CSF protein profiles. We identified IGFBP3 as significantly downregulated in PDD CSF, validated this finding via ELISA, and demonstrated its neuroprotective function *in vitro* against 1 mM MPP^+^-induced toxicity and apoptosis in SH-SY5Y cells. Mechanistically, IGFBP3 appears to exert its effects, at least partially, by activating the PI3K/Akt/GSK3β signaling pathway and modulating apoptosis regulators. These results suggest IGFBP3 as a promising candidate biomarker for PDD, potentially aiding in early diagnosis. Further investigation, particularly *in vivo* validation, is necessary to confirm its diagnostic utility and therapeutic potential.

## Data Availability

The datasets presented in this study can be found in online repositories. The names of the repository/repositories and accession number(s) can be found in the article/supplementary material. The mass spectrometry proteomics data have been deposited to the ProteomeXchange Consortium via the iProX partner repository with the dataset identifier PXD069366.
